# Risk factors for bronchial acquisition of resistant Gram-negative bacteria in critically ill patients and outcome

**DOI:** 10.1186/cc10652

**Published:** 2012-03-20

**Authors:** I Papakonstantinou, E Perivolioti, C Vrettou, I Baraboutis, E Magira, E Balioti, D Panopoulou, T Pitsolis, C Routsi, S Nanas

**Affiliations:** 1 Evaggelismos Hospital, Athens, Greece; 2 National and Kapodistrian University of Athens, Greece

## Introduction

It has been advocated that resistant Gram-negative bacteria (RGNB) colonization of ICU patients is to some extent a result of increased use of antibiotics. The aim of our study was to investigate, in adjustment with patients' characteristics, the impact of colonization status and antibiotic use during ICU stay on the impending acquisition of RGNB in the bronchial tree of newly intubated patients and to estimate the outcome.

## Methods

Bronchial and pharyngeal surveillance cultures were obtained up to day 7 (d7) of ICU admission. RGNB considered for analysis on d7 were *A. baumannii *(RAB) and *K. pneumoniae *(RKP). Polymicrobial colonization with ≥2 RGNB (PMC) was also evaluated. To assess dependence between different explanatory variables, multivariable logistic regression was used. Variables included in the model were: SOFA score, department prior to ICU admission, medical cause of admission, emergency surgery, CRF, prior aminoglycosides and tigecycline use during ICU stay and concurrent RAB or RKP pharyngeal colonization, respectively. To estimate outcome (death), variables included in multivariate model were: APACHE, SOFA score, department prior to ICU admission, medical cause of admission, emergency surgery, CRF and d7 RAB, RKP.

## Results

Ninety-five eligible patients with bronchial colonization data on d7 were included for further analysis. In the case of RAB in multivariate model (*R*^2 ^= 0.538), pharyngeal d7 RAB was the only predictor of d7 RAB bronchial colonization (OR 0.042, 95% CI 0.012 to 0.148, *P *< 0.001). In the case of RKP in multivariate model (*R*^2 ^= 0.648), pharyngeal d7 RKP (OR 0.037, 95% CI 0.004 to 0.031, *P *= 0.004), aminoglycoside use (OR 0.094, 95% CI 0.015 to 0.573, *P *= 0.01) and SOFA score (OR 1.66, 95% CI 1.07 to 2.58, *P *= 0.023) characterized bronchial d7 RKP colonization. Multivariate model for PMC (*R*^2 ^= 0.49) revealed only d7 pharyngeal PMC as predictor of bronchial PMC (OR 0.12, 95% CI 0.026 to 0.50, *P *= 0.004). Department prior to ICU, medical cause of admission, CRF, and emergency surgery were not found to influence RGNB bronchial colonization. Outcome death increased with APACHE score (OR 0.84, 95% CI 0.76 to 0.94, *P *= 0.002) and bronchial d7 RKP colonization (OR 9.14, 95% CI 1.3 to 64.4, *P *= 0.026).

## Conclusion

Of the parameters included in our model, concurrent d7 pharyngeal RAB and RKP, respectively, resulted eventually in bronchial colonization with the same pathogens. Of the overall antibiotics used only aminoglycosides had significant correlation only for RKP colonization.

